# Population genetics and phylogenomic insights into the origin of economically important black pepper (*Piper nigrum*)

**DOI:** 10.1002/ajb2.70187

**Published:** 2026-04-09

**Authors:** Dominik Metschina, Luiz A. Cauz‐Santos, Maarten J. M. Christenhusz, Nilni A. Wimalarathna, Tara D. Silva, Andreas Berger, James W. Byng, Harsha Dissanayake, Deepthi Yakandawala, Anushka M. Wickramasuriya, Barbara Turner, Mark W. Chase, Rosabelle Samuel

**Affiliations:** ^1^ Department of Botany and Biodiversity Research University of Vienna Rennweg 14 Vienna 1030 Austria; ^2^ Hortus Botanicus University of Technology Delft 2628 BM Delft The Netherlands; ^3^ Department of Plant Sciences University of Colombo Colombo 00300 Sri Lanka; ^4^ Natural History Museum Burgring 7 Vienna 1010 Austria; ^5^ Department of Export Agriculture Central Research Station Matale Sri Lanka; ^6^ Department of Botany, Faculty of Science University of Peradeniya Peradeniya Sri Lanka; ^7^ Department of Ecosystem Management, Climate and Biodiversity BOKU University Gregor‐Mendel Strasse 33 Vienna 1180 Austria; ^8^ Royal Botanic Gardens, Kew Richmond TW9 3DS Surrey UK

**Keywords:** Allopolyploidy, autopolyploidy, black pepper, origins of crop species, phylogenomics, *Piper nigrum*, Piperaceae, Sri Lankan endemics

## Abstract

**Premise:**

We conducted population genetic and phylogenomic analyses of several cultivated *Piper nigrum* varieties and closely related species. We sought to establish (1) the genetic constitution of *P. nigrum* and its putative status as an allotetraploid hybrid of Indian origin, and (2) its relationships to other species of *Piper* in South and Southeast Asia.

**Methods:**

We analyzed high‐quality data comprising single nucleotide polymorphisms (SNPs) using RAxML, a phylogenetic network approach, and coancestry analyses to examine phylogenetic relationships. We included two putative parental species of the hypothesized allotetraploid *P. nigrum* (*P. galeatum* and *P. trichyostachyon*) and a set of Sri Lankan endemic species. We also determined genome sizes of several species.

**Results:**

A clade comprising Sri Lankan endemics is more closely related to cultivated *P. nigrum* than the two Indian species. Most cultivated varieties of *P. nigrum* are autotetraploids that can be distinguished genetically, but among them are some diploid accessions.

**Conclusions:**

*Piper nigrum* is a member of a clade of Sri Lankan endemics and did not originate via hybridization between the previously suggested parental species, *P. galeatum and P. trichostachyon*. Autotetraploid varieties of *Piper nigrum* are predominant in cultivation, probably due to their increased vigor and higher yields, although diploid varieties are also present, demonstrating that genome duplication probably occurred recently in cultivation.

*Piper nigrum* L. (Piperaceae) is the source of black pepper, one of the oldest and most widely traded spices globally. Based on knowledge of historical agricultural practices, trade networks and morphological similarities, *P. nigrum* has been hypothesized to have originated in the humid tropical evergreen forests of the Malabar Coast, Western Ghats, as an allotetraploid hybrid between indigenous Indian species (Ravindran, 1990; Krishnamoorthy and Parthasarathy, 2010; Sen et al., 2019), but much of this history is speculative due to the antiquity of its cultivation. The Malabar Coast stretches from Konkan to Kanyakumari and includes one of the wettest regions of the subcontinent. Until the thirteenth century, nearly all traded black pepper came from Peninsular India. Reid (1990) mentioned the inaugural state‐sponsored trading mission led by Admiral Zheng He (Cheng Ho) in 1405 as the starting point for the Southeast Asian commercial era, facilitating the introduction of Indian pepper plants to northern Sumatra, subsequently catalyzing a significant expansion in pepper production for the Chinese market. Pepper cultivation quickly expanded, particularly along the west coast of Sumatra, a region that remained largely beyond Portuguese control, unlike the Malabar Coast where most of the Portuguese naval forces were deployed.

The Portuguese also introduced the crop to Nigeria during their virtual monopoly on the spice trade after discovery of the sea route around the Cape of Good Hope in 1498 (Gentry, 1955). By the early seventeenth century, with the arrival of Dutch and English vessels, black pepper had already become a prominent cash crop in the Indonesian islands of Sumatra and Java (Keay, 2006). Highly productive varieties of *P. nigrum* were introduced as a crop in tropical and subtropical regions, so that today it is grown commercially in many countries (Krishnamoorthy and Parthasarathy, 2010). Export values in 2020 have been reported (Paul, 2023) for Vietnam (250,000 tons), Brazil (78,000 tons), Indonesia (78,000 tons), India (104,071 tons), Sri Lanka (21,800 tons), and Malaysia (24,000 tons).

*Piper* species occurring in the wild are usually dioecious and include both diploids and tetraploids. Cultivated *P. nigrum* accessions are monoecious with self‐pollinating bisexual flowers and are mostly reproduced by cuttings. Higher yielding forms have a greater percentage of bisexual flowers, often more than 80%, so that fruit production is maximized.

Today, there are many varieties of pepper bred in India such as Karimunda, Kottanadan, Panniyur‐1, Panniyur‐3, Panniyur‐4, Panniyur‐5, PLD‐2, Subhakara (Krishnamoorthy and Parthasarathy, 2010). Although Sri Lanka produces less black pepper, it is reputed to grow the finest, characterized by high levels of piperine (the pungent component; Jansz et al., 1983; Putalun and De‐Eknamkul, 1993; Liyanage, 2010). Sri Lanka possesses a rich genetic diversity of *P. nigrum* (Wimalarathna et al., 2024), with the cultivated black pepper germplasm collection of Sri Lanka comprising various local selections as well as Panniyur‐1 from India and Malaysian Kuching introduced in 1970 (Samuel et al., 2022).

The basic chromosome number in *Piper* is *x* = 13, and cultivated varieties of *P. nigrum* are *2n* = 52, which in *Piper* is tetraploid (Mathew, 1958, 1973). Polyploidy was involved in the ancestry of many *Piper* species in India (Mathew et al., 1999). Morphological assessments of *P. wightii* Miq., *P. trichostachyon* (Miq.) C. DC. and *P. galeatum* (Miq.) C DC., all *2n* = 52, have concluded that some of these are likely to have been the parents of allotetraploid *P. nigrum*, also 2*n* = 52 (Ravindran, 1990). However, no genetic studies have been carried out to verify this. *Piper galeatum* has also been reported as 2*n* = 40 (Bai and Subramanian, 1985) and 2*n* = 52 (Mathew et al., 1999). *Piper trichostachyon* is so like *P. nigrum* in its fruit morphology that it is sometimes used as an adulterant for *P. nigrum* fruits (Rahiman and Nair, 1987).

Here, we investigated the origin of *Piper nigrum* (black pepper) using next‐generation target capture sequencing (Johnson et al., 2019) including accessions of commercial cultivars of *P. nigrum*, such as Panniyur (9), Kuching (8), and several varieties of unknown origin (21). We also included accessions of closely related species from India (2) and Sri Lanka (7). An advantage of target capture is being able to include herbarium specimens, which often have degraded DNA (Chung et al., 2016). This technique has been used in many phylogenetic studies, but the single nucleotide polymorphisms (SNPs) in the loci produced by this technique have proven useful in population genomic studies, enabling evaluation of key demographic parameters such as ploidy, heterozygosity, and introgression (Choo et al., 2020; Slimp et al., 2021; O'Connell et al., 2022).

We included 38 *P. nigrum* accessions from seven countries, encompassing both named and anonymous cultivars, which should permit us to gain insights into the origins of these varieties and thus serve as a framework to focus future studies. Our main objectives are twofold: (1) to determine the genetic constitution of *P. nigrum* and assess its ploidy and postulated hybrid origin and (2) to clarify its relationships with other *Piper* species of South and Southeast Asia.

## MATERIALS AND METHODS

### Taxonomic sampling and DNA extraction and sequencing

We obtained plant material from various herbaria, botanical gardens, Sri Lankan home gardens, and the wild (Appendix S1): India (2 outgroup species + 10 Indian selections of *P. nigrum* of unknown varieties cultivated at the Central Research Station, Matale; and the Royal Botanical Gardens, Peradeniya, Sri Lanka), Sri Lanka (21), Indonesia (1), Cambodia (1), Malaysia (3), Thailand (2), China (4), and anonymous (3). Before selecting this final set of samples, we ran a preliminary maximum‐likelihood analysis with broader species coverage (Appendix S2) including 155 accessions, among these all accessions used by Metschina et al. (2025). Our main sampling strategy was to include material, especially cultivated *P. nigrum* from several pepper‐growing countries, to determine the genetic background of wild and commercial varieties. We also included two, *P. trichostachyon* and *P. galeatum*, of the three species suggested previously on morphological grounds to be parents of *P. nigrum*.

From samples of silica‐gel‐dried, freeze‐dried, and herbarium leaves, up to 50 mg of leaf material was frozen in liquid nitrogen and ground using a TissueLyser II (Qiagen, Hilden, Germany). Total genomic DNA was extracted using the DNeasy Plant Mini Kit (Qiagen) with slight modifications, including increasing AP1 buffer volumes from 400 µL to 500 µL and an additional step using chloroform–isoamyl alcohol (24:1). NEBNext Ultra II DNA Libraries (New England Biolabs, Ipswich, MA, USA) were prepared following the manufacturer's instructions (version 6.1_5/20) using half volumes of reagents. For each sample, up to 400 ng of input DNA was used in 100 µL ddH_2_O and sheared using a Bioruptor Pico sonication device (Diagenode, Liège, Belgium). The shearing settings for high‐molecular DNA to target a mean insert size of ca. 350 bp were 15 s on, 90 s off; seven cycles at 4°C. For DNA extracted from herbarium specimens, no shearing was carried out. NEBNext Multiplex Oligos for Illumina (96 Unique Dual Index Primer Pairs) were used. After library prep, the DNA samples were sorted according to their DNA quantity, ranging from highest to lowest and by sample quality/DNA degradation (fresh or herbarium material). They were pooled in sublibraries, each containing a maximum of 16 samples with unique dual index primer pairs. These sublibraries were used as input for target enrichment, which was performed using an Angiosperms353 probe kit (Johnson et al., 2019; available from Arbor BioScience, Ann Arbor, MI, USA), following the user manual version 5.0. The libraries were then sequenced as paired‐end reads of 150 bp.

### Genome size

Genome size was assessed using fresh leaf samples and material stored at –80°C. We included *P. zeylanicum* Miq., *P. trineuron* Miq., and two cultivated accessions of *P. nigrum*. The leaf material was chopped and then stained with CyStain PI OxProtect (Sysmex Partec, Münster, Germany) according to the manufacturer's instructions, and genome sizes were estimated via flow cytometry on a CyFlow Space cytometer (Sysmex Partec). *Solanum pseudocapsicum* L. served as the reference standard. Because *S. pseudocapsicum* and *P. zeylanicum* exhibited similar genome sizes, the genome size of the latter was estimated based on that of *P. walkeri*, which was used as a reference in this case.

### Bioinformatics

Demultiplexing, removal of adapters, and quality control (FastQC and MultiQC) was performed by the Next Generation Sequencing Facility at the Vienna BioCenter Core Facilities, Austria. The cleaned data were processed further by mapping the reads to the reference genome of *P. nigrum* (Hu et al., 2019) using BWA (Li and Durbin, 2009). The reads were sorted by position in the reference genome using SAMtools (Li et al., 2009). The Picard toolkit was used to annotate the sorted SAM files with read groups, using the command AddOrReplaceReadGroups. Variants were called with GATK4 (McKenna et al., 2010), following the programs best‐practices recommendations. First, HaplotypeCaller was used to generate intermediate gVCF files for each sample, set either to diploid or tetraploid, and using the ‐ERC GVCF mode, to allow for subsequent joint genotyping. In the following steps performed with GATK4, the data were split into 26 genomic intervals, corresponding to 26 chromosomes, using the *‐L* flag. All single‐sample GVCF‐files were imported into a GenomicsDB, using the GenomicsDBImport tool. Joint genotyping of each ploidy data set (diploid and tetraploid) was then performed using the GenotypeGVCFs module. GatherVCFs was used to merge all 26 genotyped VCF files for both data sets. After merging, the resulting VCF file was filtered using the VariantFiltration module of GATK with the following criteria: (1) quality by depth (QD) <2.0; (2) Phred‐scaled *P*‐value for Fisher's exact test to detect strand bias >60; and (3) a root mean square of mapping quality across all samples (MQ) <40. After these steps, bcftools isec was used to create an intersect of positions present in both the diploid and the tetraploid data set. Only sites which were present in at least 80% of the samples and only biallelic SNPs were retained, using bcftools v1.21 (Danecek et al., 2021).

PolyRelatedness 1.11b (Huang et al., 2014) was used to evaluate pairwise relatedness between samples by generating a coancestry heatmap from a relatedness coefficient matrix, using a coancestry estimator based on Ritland (1996) suitable for mixed ploidy data sets. We implemented PolyRelatedness among accessions of *P. nigrum* and close relatives via covariance matrices calculated based on genotypes called in GATK. We reduced the data set due to limitations of the program PolyRelatedness, selecting 50,000 SNPs with the least missing data using a custom script. The heatmap of coancestry was visualized with the function heatmap.2 implemented in the R package Gplots version 3.3.0 (Warnes et al., 2025).

Genotypes were also used to select unlinked sites with 10,000 bp between variants, producing a file of 11,256 genotypes, which were then extracted from the VCF file and formatted to the input file format of the program structure (Pritchard et al., 2000). structure was run with *K* (number of genetic clusters) from 1 to 10, with 10 replicates per *K*, a burn‐in of 200,000 and 1000,000 repetitions per individual run. For summarization and visualization of the different clustering models (Ks), we used Clumpak (Kopelman et al., 2015) and distruct (Rosenberg, 2004). The output was edited graphically using Inkscape (Inkscape Project, 2024).

For phylogenomic analyses, after we removed singletons and sites with more than 20% missing data, we converted the VCF file to PHYLIP file format. Ascertainment bias correction was performed with the python script ascbias.py (https://github.com/btmartin721/raxml_ascbias). For maximum likelihood analyses, RAxML 8.2.12 (Stamatakis, 2014) was used with the –asc‐corr=lewis and ‐m ASC_GTRCAT command, applying the Lewis ascertainment bias correction and using the general time reversible model of nucleotide substitution and the CAT approximation of rate heterogeneity, performing a search for the best‐scoring maximum likelihood tree with 1000 rapid bootstrap replicates. The outgroup was specified as the two Indian species based on the heatmap results. Phylogenetic network analyses were carried out with SplitsTree (Huson and Bryant, 2006), using the VCF file before ascertainment bias correction, containing 921,085 SNPs.

For assessing the mode of inheritance (tetrasomic vs. disomic) in cultivated *P. nigrum*, a custom python script (available at https://github.com/DomiMet/allo_or_autotetraploid.git) was used, plotting allele frequencies against genotype frequencies obtained from the filtered VCF file.

## RESULTS

### Preliminary analysis

We performed a preliminary RAxML analysis with 300 bootstrap replicates of a 1,790,008‐SNP matrix that also incorporated the accessions from Metschina et al. (2025) to identify taxa closely related to *P. nigrum* (Appendix S2). The data preparation and filtering were performed as described in the Materials and Methods. Overall, the tree was well resolved with high support (bootstrap percentage [BP] ≥98) for all nodes at interspecific levels. Although 10 species that are either found in or are endemic to India were sampled, only *P. trichostachyon*, *P. galeatum*, *P. schmidtii* Hook.f., and *P. barberi* Gamble were related to *P. nigrum*, and most of the other Indian species were in a more distantly related clade. A clade comprising the Sri Lankan endemics *P. trineuron* and *P. zeylanicum* and two accessions of native *P. hymenophyllum* were sister to *P. nigrum*. For the subsequent analyses, we retained only the taxa relevant to our research questions.

## COVARIANCE MATRIX

**Figure 1:**
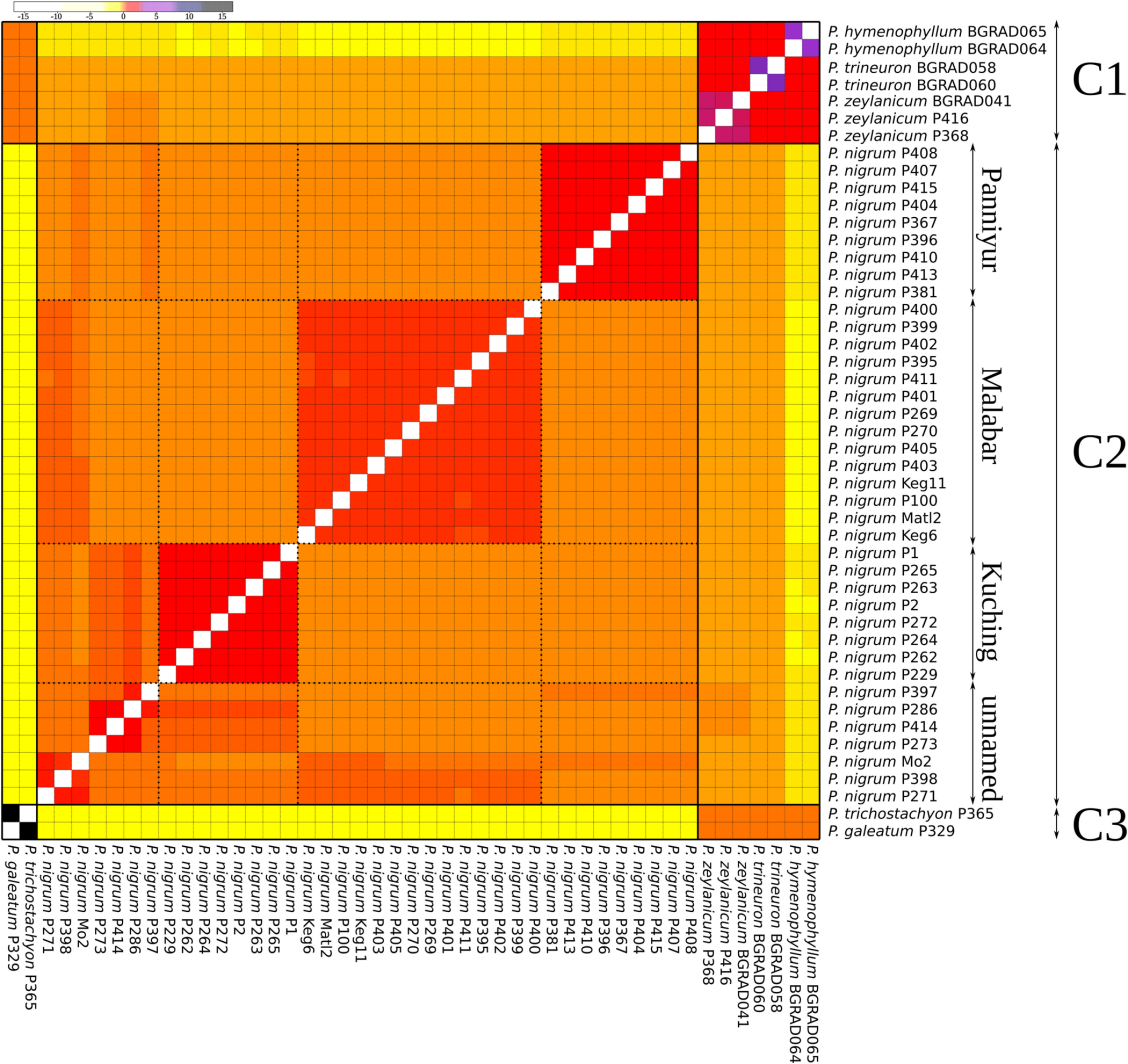
Heatmap of pairwise relatedness based on 50,000 SNPs. Legend (top right): relatedness coefficients as estimated by Ritland's method‐of‐moments estimator.

In analyses of 50,000 SNPs, we observed a distinct cluster (C1) comprising Sri Lankan endemics (*P. zeylanicum and P. trineuron*) and native but putatively more widespread *P. hymenophyllum* Miq. (Figure 1). Cluster 2 represents accessions of cultivated *P. nigrum*, which can be divided in five distinct subclusters, representing high yielding, often commercially grown varieties of black pepper, Panniyur, Kuching, Malabar, and unnamed cultivars, which can be divided in two subclusters. These exhibit a high level of relatedness to each other and less relatedness to cluster 3, *P. trichostachyon* and *P. galeatum* (*P*. sect. *Muldera*), the putative parental species of *P. nigrum*. These exhibit high pairwise relatedness to each other, lower relatedness to the Sri Lankan endemics and low relatedness to cluster 2. Accessions of the Sri Lankan endemics treated as diploids (*P. trineuron* and *P. zeylanicum*) show moderate levels of relatedness among all accessions, whereas tetraploid *P. hymenophyllum* exhibits low relatedness with C2 and moderate relatedness with C3.

### Maximum likelihood tree and phylogenetic network analysis

**Figure 2:**
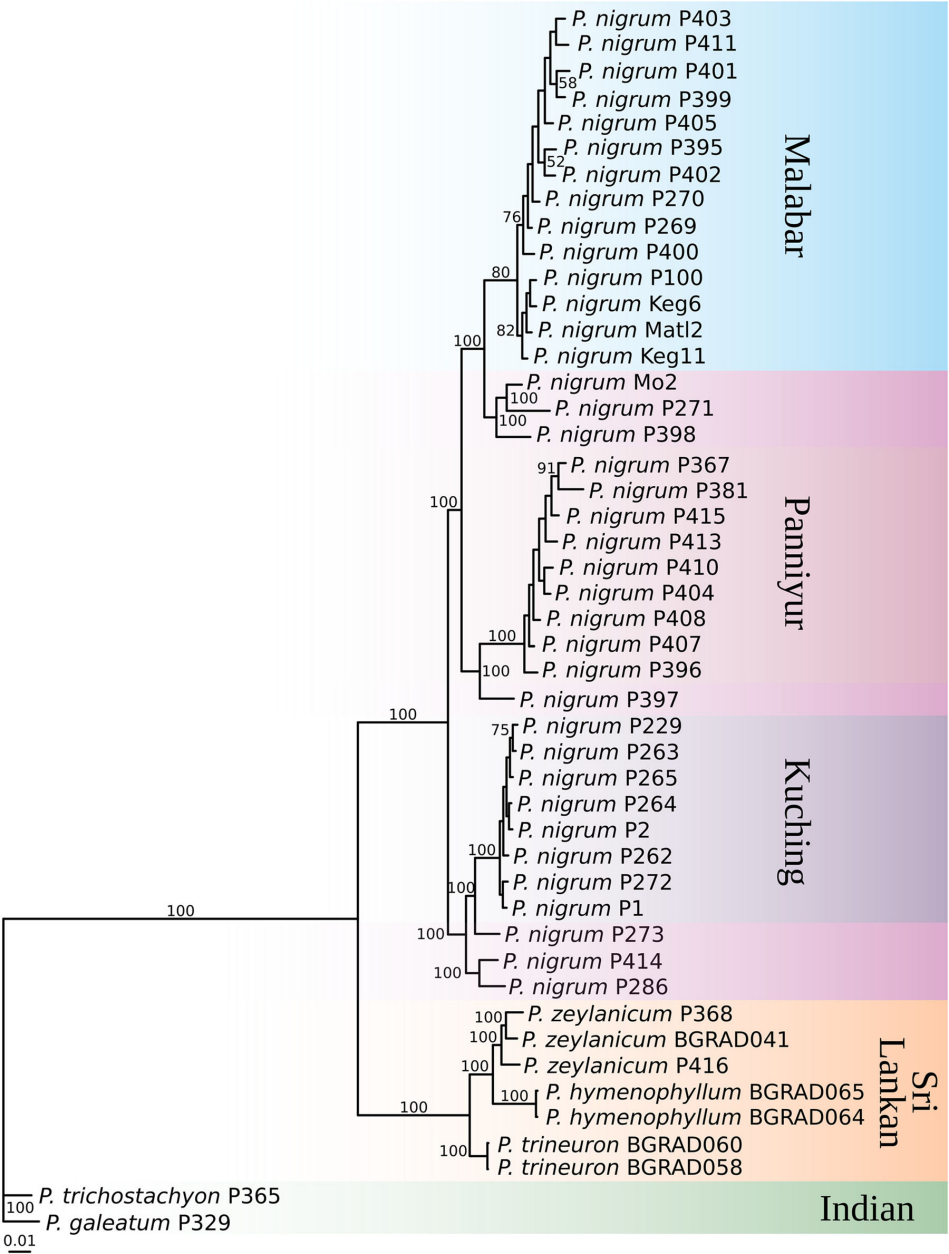
Maximum likelihood tree of *P. nigrum* and closely related species from RAxML analysis. ML tree obtained from an alignment consisting of 330,271 SNPs. Values above or near branches are bootstrap percentages (values of 50 and below are not shown). Clade names are indicated.

The RAxML tree of our final data set based on 330,271 SNPs has high support (BP 100) at interspecific levels (Figure 2). The various cultivated varieties of *P. nigrum* are collectively sister to the Sri Lankan endemics with high bootstrap support (BP 100). The putative parental species of cultivated *P*. *nigrum* (*P. trichostachyon* and *P. galeatum*) are highly supported (BP 100) and sister (BP 100) to the clade of Sri Lankan endemics (BP 100) plus *P. nigrum*. *Piper nigrum* can be subdivided into six highly supported subclusters (BP 100): Panniyur, Malabar, Kuching, and three clusters of anonymous varieties.

**Figure 3:**
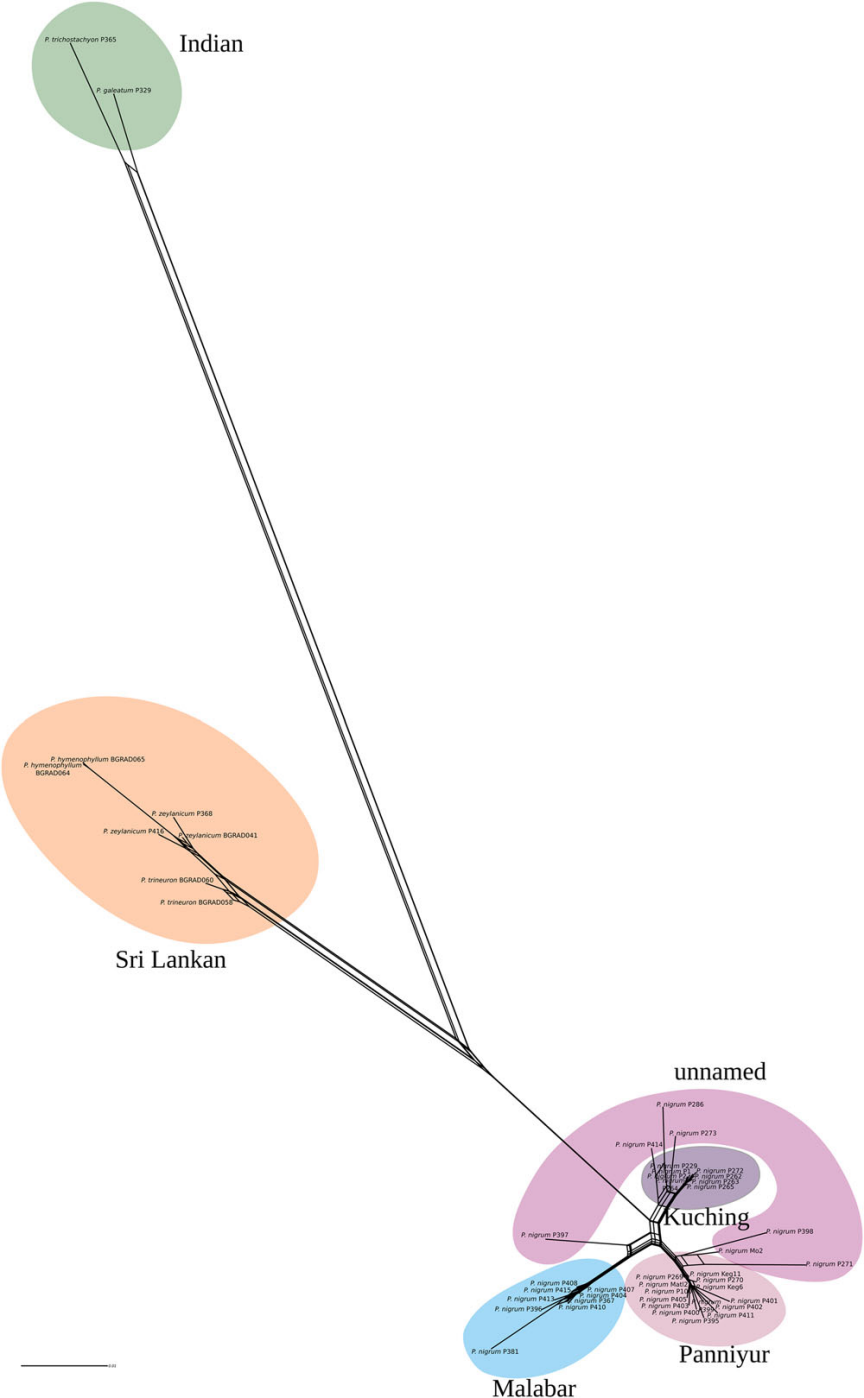
Phylogenetic network from SplitsTree analysis, obtained from an alignment consisting of 921,085 SNPs.

The matrix for the SplitsTree analysis contains 921,085 SNPs, and overall results (Figure 3) correlate well with cluster/clades in the RAxML tree. As in the heatmap, the *P. nigrum* accessions again form six clusters, Kuching, Malabar, Panniyur and three groups of anonymous cultivars. The Sri Lankan clade exhibits a degree of shared SNPs, especially among *P. trineuron* and *P. zelanicum* and *P. hymenophyllum*, which was also observed in the structure plots.

### Structure analysis

After retaining only unlinked sites with 10,000 bp between SNPs, the data set comprises 11,256 variants. In the K2 and K3 model (Appendix S3), accessions of Sri Lankan natives (all endemic, except *P. hymenophyllum*, but see Discussion) cluster with the putative parents of *P. nigrum*, *P. trichostachyon*, and *P. galeatum*, exhibiting some introgression from *P. nigrum* (blue, Appendix S3). In contrast, all *P. nigrum* accessions form one large cluster (blue) in K2. In K3, Panniyur forms a cluster on its own (purple), exhibiting introgression with accessions from the anonymous varieties, which are all diploid (see Discussion).

**Figure 4:**
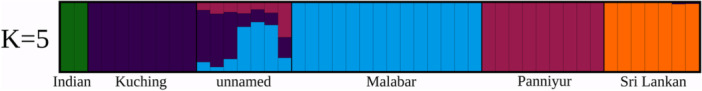
Summarized structure results of major model of *K* = 5.

In the model *K* = 5 (Figure 4), separate clusters are identified for the Sri Lankan accessions (orange), the putative parental species from India (green), Malabar selections (blue), Panniyur (magenta), Kuching (purple), and the anonymous varieties, showing introgression from all other three *P. nigrum* clades. At *K* ≥ 6, the clusters were not subdivided any further, and putative ghost introgression signals emerged.

### Genome size estimation

Both cultivated accessions of *P. nigrum* (Kuching and Panniyur) displayed an average 1 C value of 0.86 pg (Table 1). The *P. trineuron* accession had a similar genome size, 0.84 pg. *Piper zeylanicum* had the largest genome, 1.26 pg.

**Table 1:** Genome size estimation with flow cytometry.

Sample	Peak (sample)	Peak (standard)	1C sample (pg)	Mean 1C (pg)
*P. nigrum* (Kuching)	1	1.506	0.863	0.86
	1	1.504	0.864	
*P. nigrum* (Panniyur)	1	1.508	0.862	0.86
	1	1.508	0.862	
*P. trineuron*	1	1.546	0.841	0.84
	1	1.550	0.839	
*P. zeylanicum*	1.885	1*	1.261	1.26
	1.891	1*	1.265	
*P. walkeri*	1	1.942	0.670	0.67
	1	1.945	0.668	

*Solanum pseudocapsicum* was used as the standard (1C = 1.3 pg), except for cases marked with an asterisk.

* *P. walkeri* was used as the standard.

### Mode of inheritance

Based on allele frequencies plotted against genotype frequencies under the Hardy–Weinberg model (Appendix S4), tetrasomic (autotetraploid) inheritance was observed for all cultivated *P. nigrum*, except for the anonymous accessions, which appeared to be diploid (Appendix S5).

## DISCUSSION

**Figure 5:**
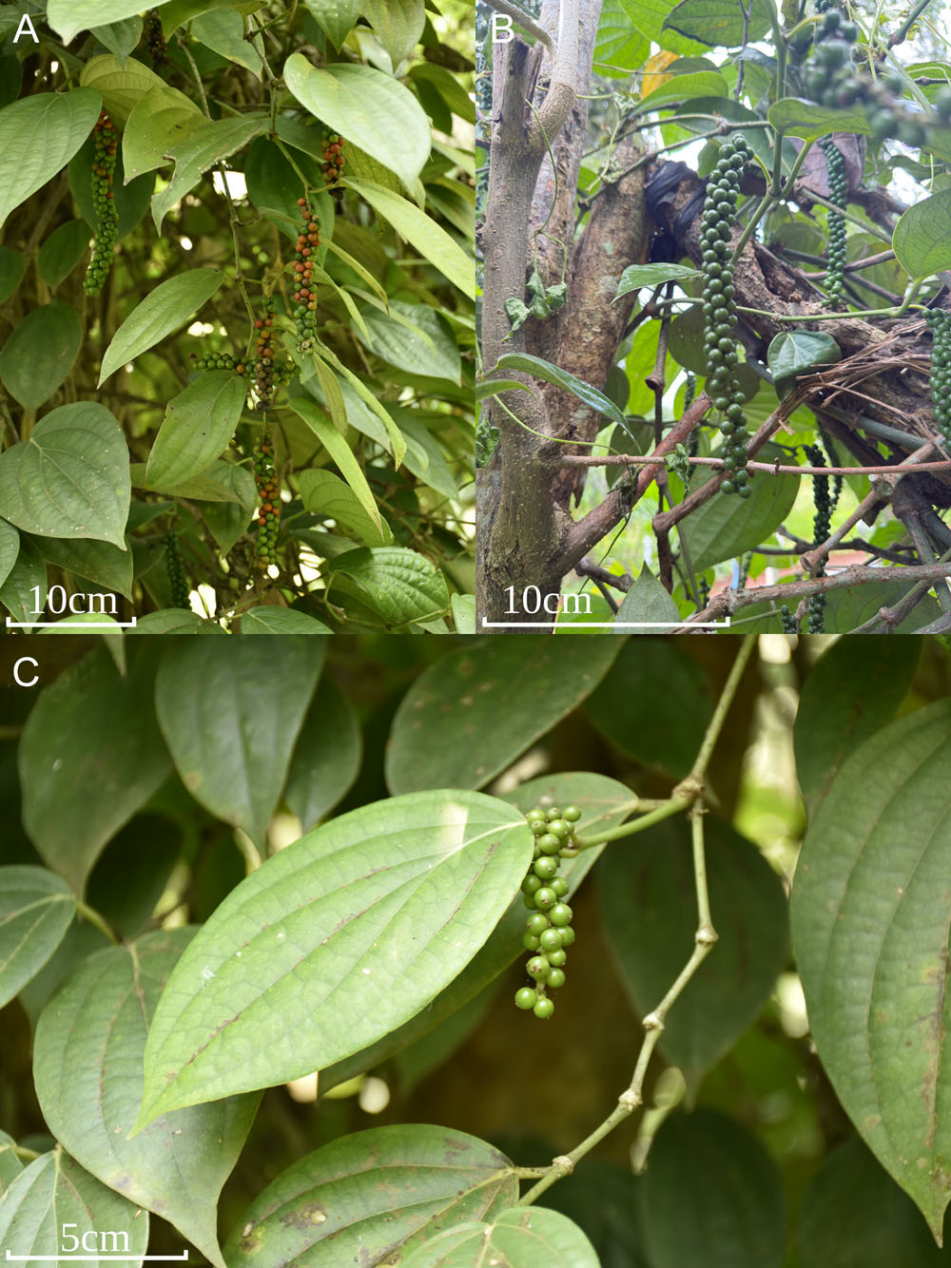
Selection of *Piper nigrum* varieties: (A) Sri Lankan local selection GK49, (B) Panniyur, (C) Kuching.

Our results show that different varieties of black pepper, such as Panniyur, Kuching, and others, can be distinguished using SNP data in the relatedness matrix and RAxML, network and structure analysis (Figures 1, 2, 3, 4). There is support for a clear population structure in cultivated *P. nigrum* based on the results of the model of K5 (structure analysis; Figure 4), which is also reflected in its morphological differences (Table 2, Figure 5), which were described in detail by Samuel (1984). All accessions of *P. nigrum* share a common genetic background in K2 (Appendix S3). In the structure results (K2 and K3; Appendix S3), the Sri Lankan endemics and *P. trichostachyon* + *P. galeatum* (*P*. sect. *Muldera*) cannot be distinguished. Given that structure tends to be more sensitive to recent admixture events, it may fail to detect signals of ancient coancestry among these taxa in higher Ks (Pang and Zhang, 2025). Therefore, we conclude that a combination of population structure in K2 and K5 might reflect best the relationships of *Piper*, capturing both deep coancestry (K2 and K3) and more recent common ancestry (K5).

**Table 2:** Morphological differences of varieties of *P. nigrum*, from Samuel (1984).

Varieties	Spike length (cm)	Internodal length (cm)	Total number of nodes	Leaf area (cm²)
Panniyur‐1	14.4	7.83	20.25	102.34
Kuching	6.24	5.80	40.25	55.85
GK49	13.75	3.99	69	73.55

Numbers represent mean of 30 individuals (2 plots,15 individuals per plot).

Hu et al. (2019) reported a whole‐genome duplication (WGD) in *P. nigrum* at roughly 17.2–17.9 million years ago (Ma), which must have occurred in the crown‐group of the *P. nigrum* clade, estimated as 14.7–18.3 Ma (Gopallawa et al., 2025) and 16.6–17.4 Ma (Metschina et al., 2025), associated with climatic shifts during the Middle Miocene Climatic Optimum, as reported for other plant species (Yamanda and Yamanda, 2018). This event likely represents an ancient WGD in the *P. nigrum* clade and not in modern *P. nigrum*, which includes both diploid and tetraploid races. In our plots (Appendix S5), the presence of all five genotype classes at an allele frequency of 0.5 provides clear evidence of tetrasomic inheritance (Lloyd and Bomblies, 2016). In the RAxML tree (Figure 2), the diploid accessions are scattered as sisters to all three main clades of the tetraploid varieties, Kuching, Panniyur and Malabar. Therefore, the most reasonable hypothesis is that cultivated varieties of *P. nigrum* are autotetraploids that appear to have recently been formed multiple times from diploid progenitors, most likely in cultivation. Based on our results, most of the cultivated *P. nigrum* are autotetraploid (2*n* = 52) with a genome size of 1 C = 0.86 pg (Table 1). Between varieties, e.g., Kuching and Panniyur, there is no difference in genome size.

The two putative parental species of *P. nigrum*, *P. trichostachyon* and *P. galeatum*, are sister to the clade of Sri Lankan endemics plus *P. nigrum*. There is thus no evidence for *P. trichostachyon* and *P. galeatum* being the parental species of *P. nigrum*. Sen et al. (2019) proposed an Indian origin of *P. nigrum* in the Western Ghats during the Miocene, but they did not include any Sri Lankan samples. Our results clearly show a sister relationship of *P. nigrum* to the Sri Lankan endemic species and indicate potential involvement of Sri Lanka in the ancestry of *P. nigrum*. We included accessions of *P. schmidtii* and *P. barberi* in our preliminary RAxML analysis (Appendix S2), in which they are related to *P. trichostachyon* and *P. galeatum*. Given the extremely degraded herbarium DNA of these samples, we removed them from the final data set to avoid introducing anomalies. Therefore, one liability we have faced in this study was limited access to many Indian species due to permit restrictions and lack of suitable herbarium material.

Additionally, our Sri Lankan *P. hymenophyllum* is a cryptic, undescribed Sri Lankan species, endemic and not related to Indian *P. hymenophyllum* (Gopallawa et al., 2025). Expanded sampling coupled with detailed morphological analyses will be essential to provide a framework to reliably assess species distinctions, although species delimitation has proven to be challenging for some closely related species of *Piper* (Suwanphakdee et al., 2016; Asmarayani, 2023). Sri Lankan *P. hymenophyllum* formed a clade with the endemic Sri Lankan species (*P. trineuron* and *P. zeylanicum*), and, thus, *P. nigrum* is a member of a clade otherwise comprising exclusively Sri Lankan endemics, which could be interpreted as it having a Sri Lankan origin. Other evidence for this origin was also provided in Jayarathna et al. (2016) but with more limited taxonomic sampling and analysis of only nuclear ribosomal ITS. More representation of Indian species should be included to test our preliminary conclusions about a putative Sri Lankan origin of *P. nigrum*.

In the SplitsTree network (Figure 3), *P. nigrum* accessions form a distinct cluster. Rather than historical gene flow, reticulation in this cluster might reflect recent directed hybridization (Sivaraman et al., 1999; Ravindran, 2000). Domestication has therefore led to genetically homogeneous populations in the widely cultivated varieties due to artificial selection narrowing their genetic base (Khoury et al., 2022).

## CONCLUSIONS

Our results show population structure and intraspecific genetic diversity in cultivated black pepper, and it is possible to distinguish between selections/cultivars with NGS data (i.e., in maximum likelihood results). This intraspecific diversity may be a result of hybridization and/or selection in cultivation. This selective process, while enhancing specific traits such as yield, disease resistance, and quality, can lead to the loss of genetic variation present in the ancestral wild populations, which in *P. nigrum* are undocumented, but it is possible that the anonymous varieties may be more closely related to the wild ancestor, a hypothesis reinforced by their diploid status. There is evidence of admixture among some *P. nigrum* varieties, mostly likely due to hybridization in cultivation. Given that a significant proportion of our accessions are from cultivated plants, the overall genetic diversity captured in our data set may be considerably reduced relative to a data set that, ideally, would also include more accessions of Indian *P. nigrum*, especially those that have been suggested to represent putatively wild populations (Sen and Rengaian, 2022), which may be escapes from cultivation rather than truly wild.

In our study, we provide novel molecular evidence that cultivated *P. nigrum* is not an allotetraploid, but rather an autotetraploid that likely evolved several times recently from diploid ancestors. It is a member of a Sri Lankan endemic clade, including *P. trineuron*, *P. zeylanicum*, and a cryptic new Sri Lankan species, previously considered to be a member of the widespread species *P. hymenophyllum*. *Piper galeatum* and *P. trichostachyon* are not the parental species of cultivated *P. nigrum* as previously hypothesized and have no exclusive relationship to it. We suggest that *Piper nigrum* may have originated in Sri Lanka and, due to the importance of black pepper as a commodity, was taken early to the Malabar Coast and from there to Sumatra, where it first became known to Europeans, giving rise to the idea that it originated in India. We acknowledge that this conclusion is weakened by the lack of extensive Indian material in our study, but our analyses provide a framework for future studies with increased sampling, especially of Indian species and more *P. nigrum* accessions than those to which we could gain access.

## AUTHOR CONTRIBUTIONS

R.S. conceived the idea and designed the study; D.M., N.A.W., A.M.W, and B.T. produced the data; D.M. conducted all analyses. L.A.C.‐S. advised on data analysis decisions; R.S., D.M., M.W.C, L.A.C.‐S. and M.J.M.C. wrote the manuscript; T. de S. and D.Y. advised on the design of the manuscript. A.B., H.D. and J.W.B. provided material.

## Supporting information


**Appendix S1**. Taxa included in the analysis.

**Appendix S2**. RAxML supermatrix. ML tree obtained from an alignment consisting of 1,790,008 SNPs. Bootstrap percentages above 50 are shown above branches. Clade names are indicated.

**Appendix S3**. Summarized structure results of 10 runs (K1–K10).

**Appendix S4**. Hardy–Weinberg model for tetraploids with tetrasomic or disomic inheritance patterns.

**Appendix S5**. Allele frequencies vs. genotype frequencies for three varieties of cultivated *P. nigrum* and unnamed diploid accessions.

## Data Availability

The raw sequence data generated in this study are available at https://www.ncbi.nlm.nih.gov/sra/PRJNA1142735.
