# Supplementary material for: Population genetics and phylogenomic insights into the origin of economically important black pepper (Piper nigrum)
Source: Am J Bot. 2026 Apr 9;113(4):e70187. doi: 10.1002/ajb2.70187 (PMC13103621; doi:10.1002/ajb2.70187)

Appendix S2. RAxML supermatrix. ML tree obtained from an alignment consisting of 1,790,008 SNPs. Bootstrap percentages (BP) above 50 are shown above branches.

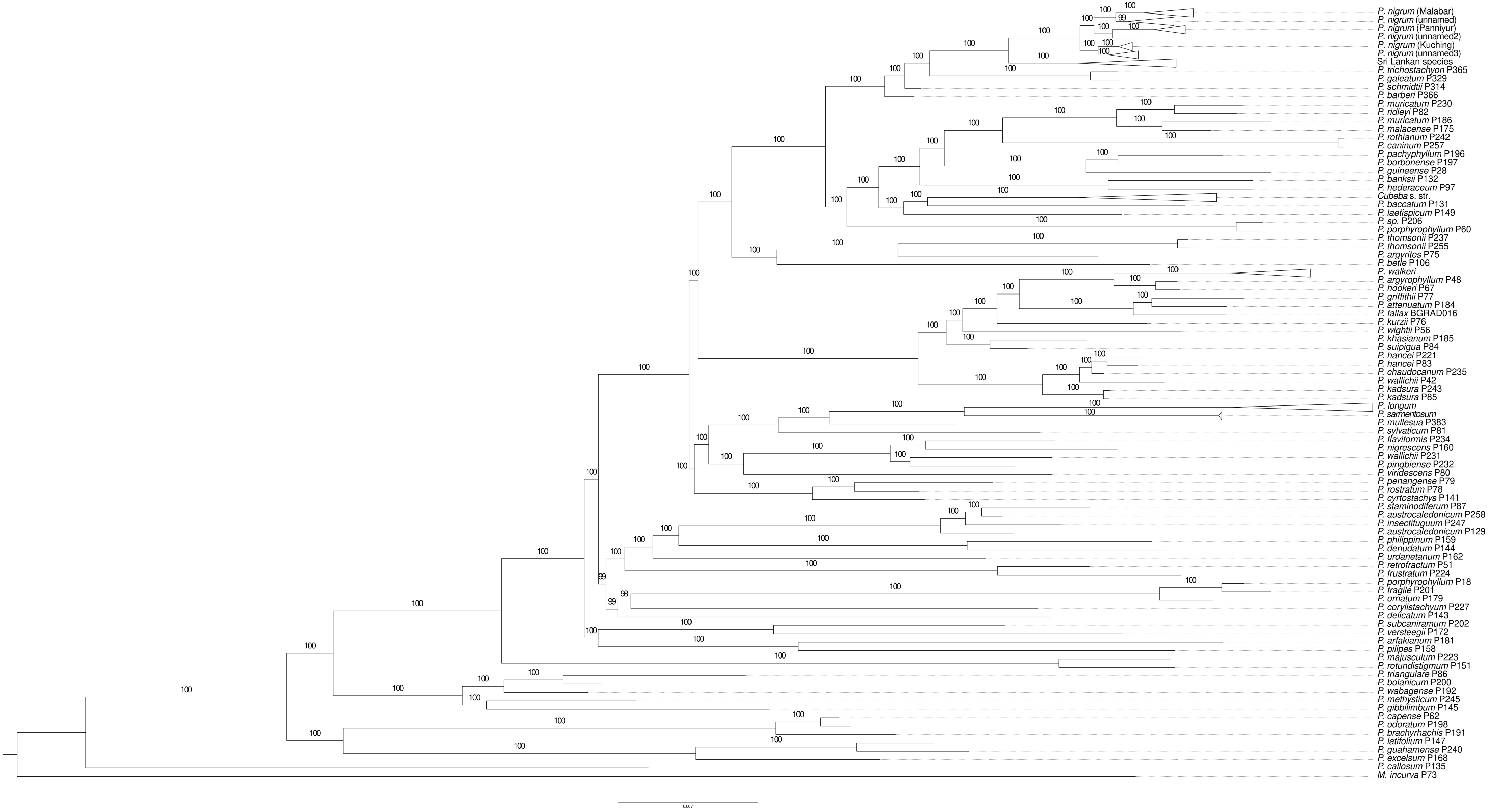

Supplement: Supplementary file 2 — Appendix S2. RAxML supermatrix. ML tree obtained from an alignment consisting of 1,790,008 SNPs. Bootstrap percentages above 50 are shown above branches. Clade names are indicated. [file AJB2-113-e70187-s001.pdf]
