# Supplementary figures and images for: Population genetics and phylogenomic insights into the origin of economically important black pepper (Piper nigrum)
Source: Am J Bot. 2026 Apr 9;113(4):e70187. doi: 10.1002/ajb2.70187 (PMC13103621; doi:10.1002/ajb2.70187)

Appendix S3. Summarized STRUCTURE results of 10 runs (K1–K10).

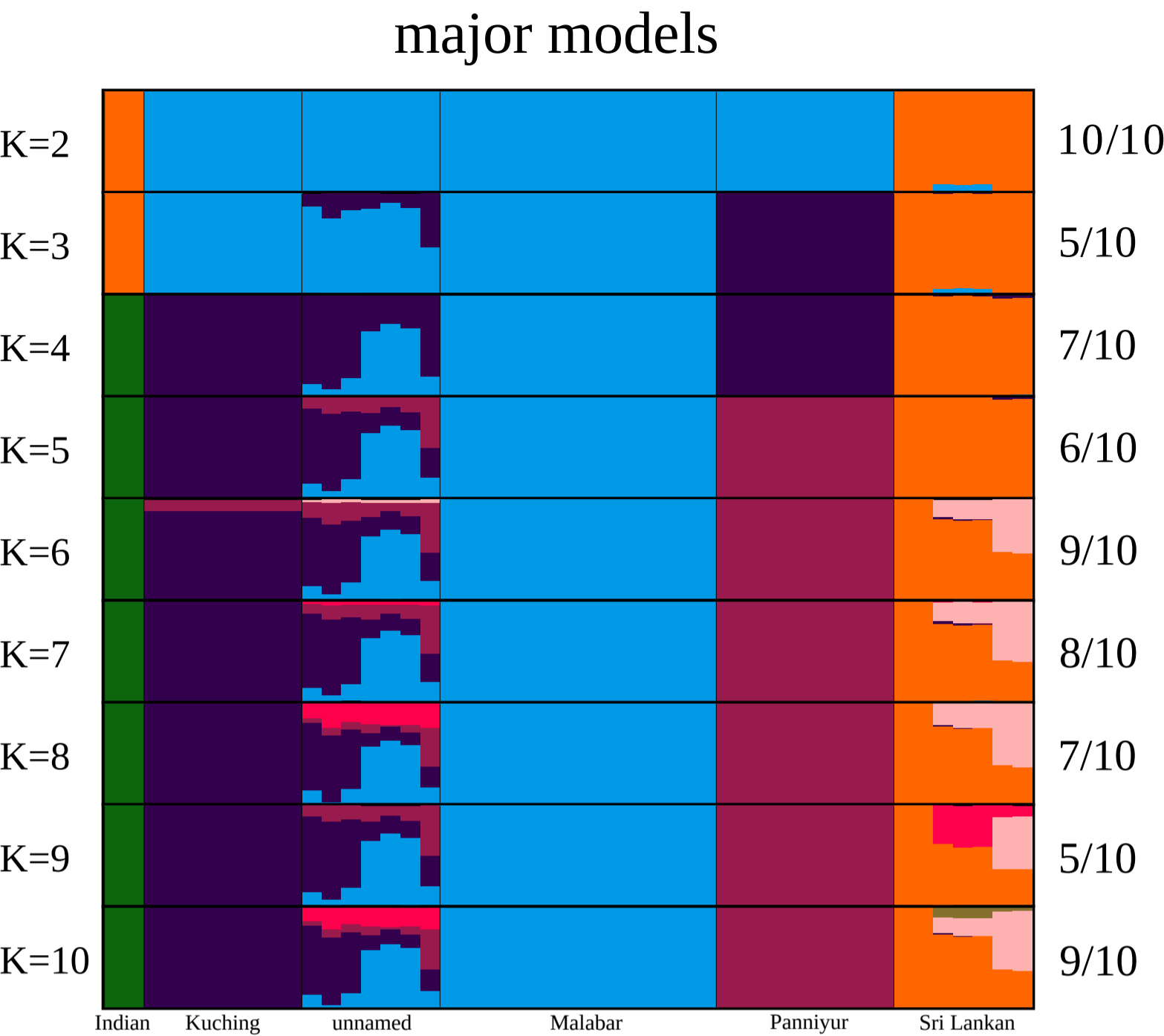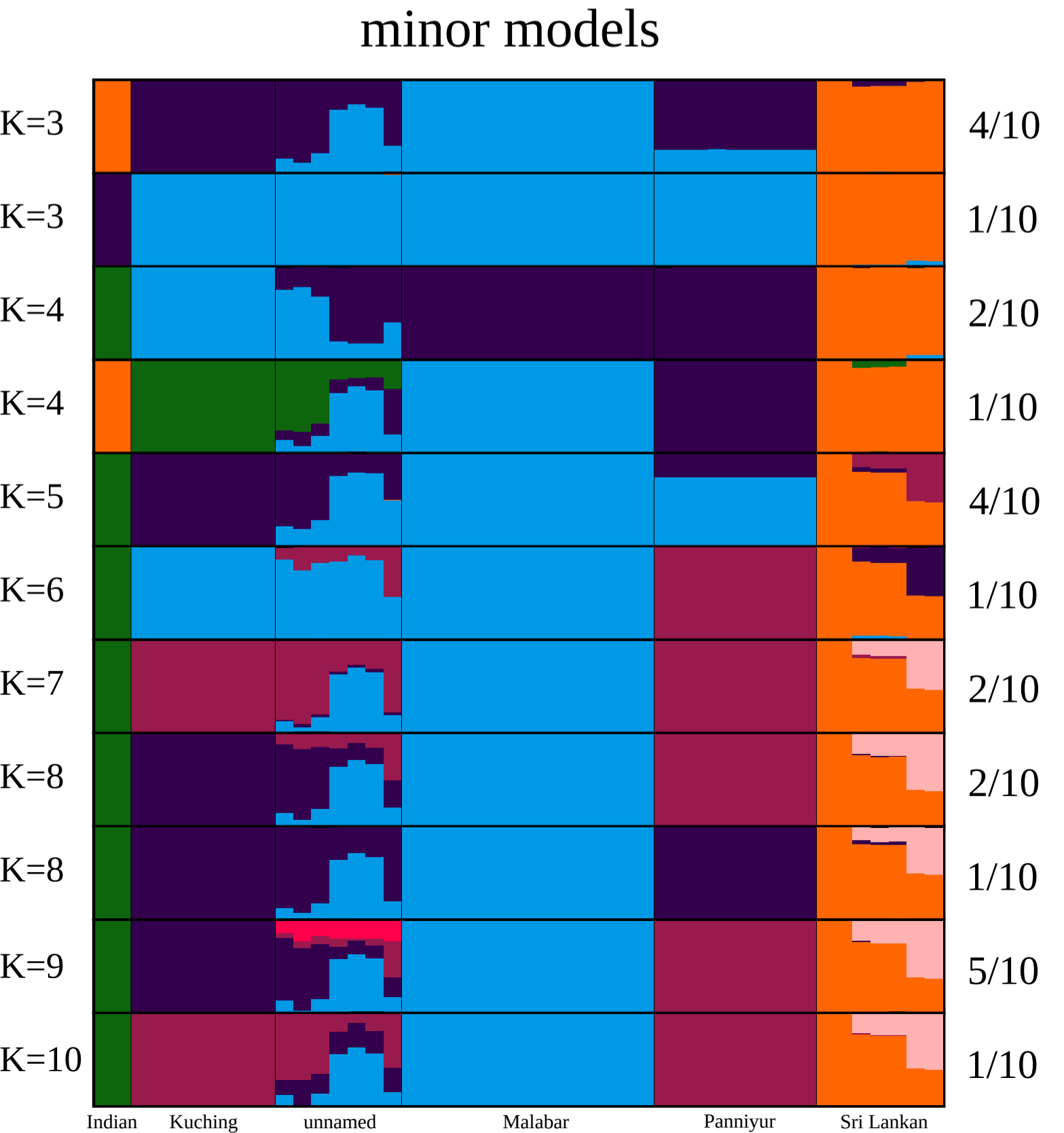

Supplement: Supplementary file 3 — Appendix S3. Summarized structure results of 10 runs (K1–K10). [file AJB2-113-e70187-s003.pdf]
