# Supplementary material for: Population genetics and phylogenomic insights into the origin of economically important black pepper (Piper nigrum)
Source: Am J Bot. 2026 Apr 9;113(4):e70187. doi: 10.1002/ajb2.70187 (PMC13103621; doi:10.1002/ajb2.70187)
Supplement: Supplementary file 4 — Appendix S4. Hardy–Weinberg model for tetraploids with tetrasomic or disomic inheritance patterns. [file AJB2-113-e70187-s005.docx]

**Appendix S4**. Hardy–Weinberg model for tetraploids with tetrasomic or disomic inheritance patterns.


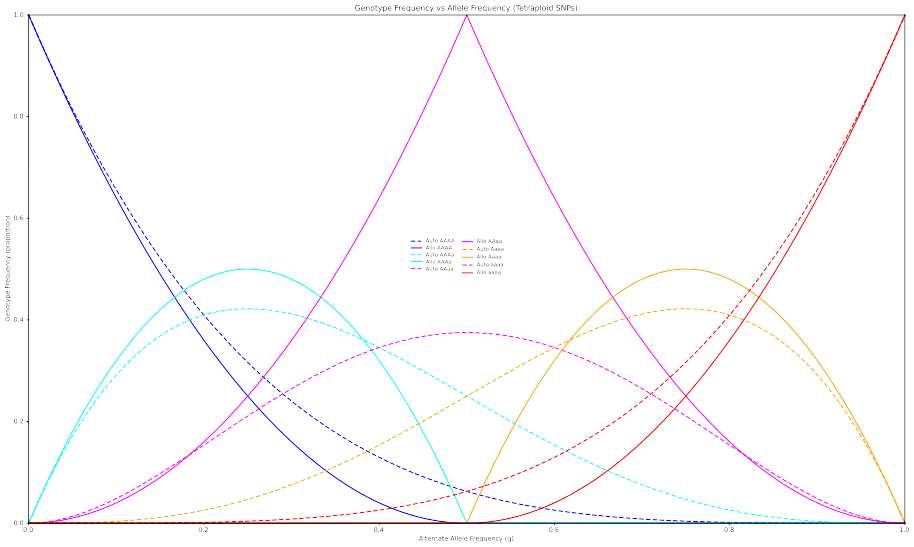
Allele frequencies vs. genotype frequencies under Hardy–Weinberg model for tetraploids with tetrasomic (autotetraploid) or disomic (allotetraploid) inheritance patterns.
