# Supplementary material for: Population genetics and phylogenomic insights into the origin of economically important black pepper (Piper nigrum)
Source: Am J Bot. 2026 Apr 9;113(4):e70187. doi: 10.1002/ajb2.70187 (PMC13103621; doi:10.1002/ajb2.70187)
Supplement: Supplementary file 5 — Appendix S5. Allele frequencies vs. genotype frequencies for three varieties of cultivated P. nigrum and unnamed diploid accessions. [file AJB2-113-e70187-s002.docx]

**Appendix S5.** Allele frequencies vs. genotype frequencies for three varieties of cultivated *P. nigrum* and unnamed diploid accessions.

Kuching

*
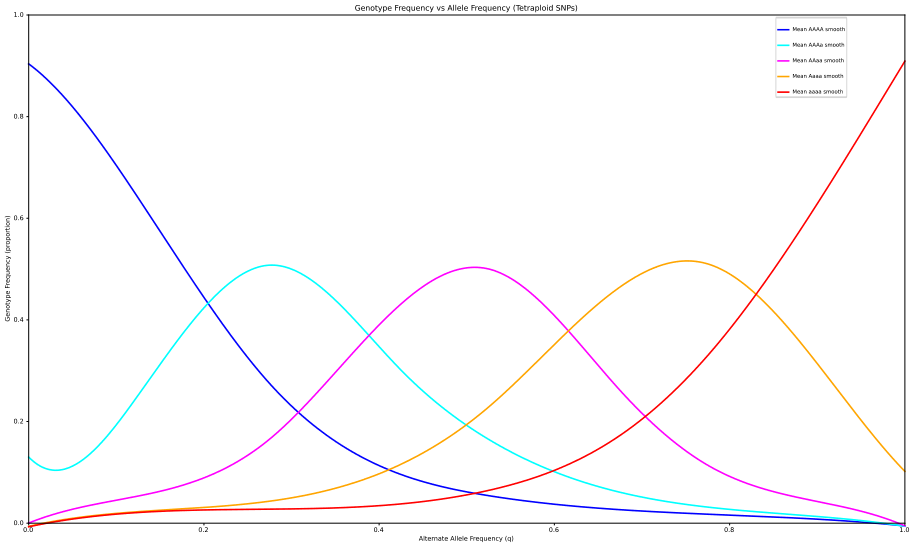
*

Panniyur

*
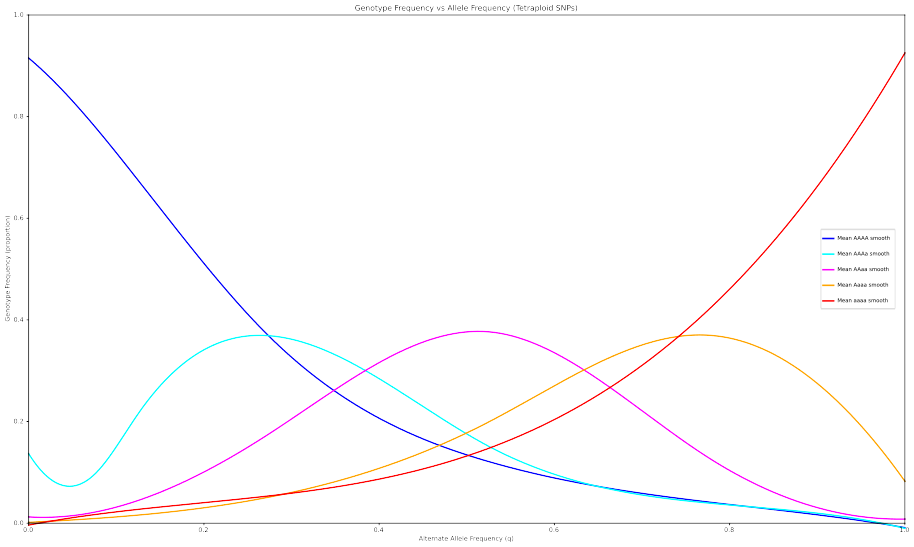
*

Malabar selections

*
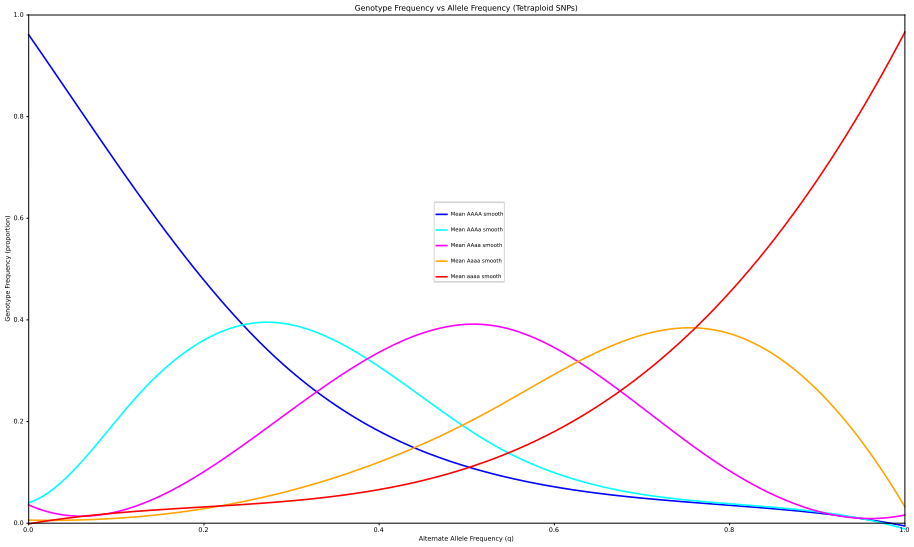
*

Unknown varieties

*
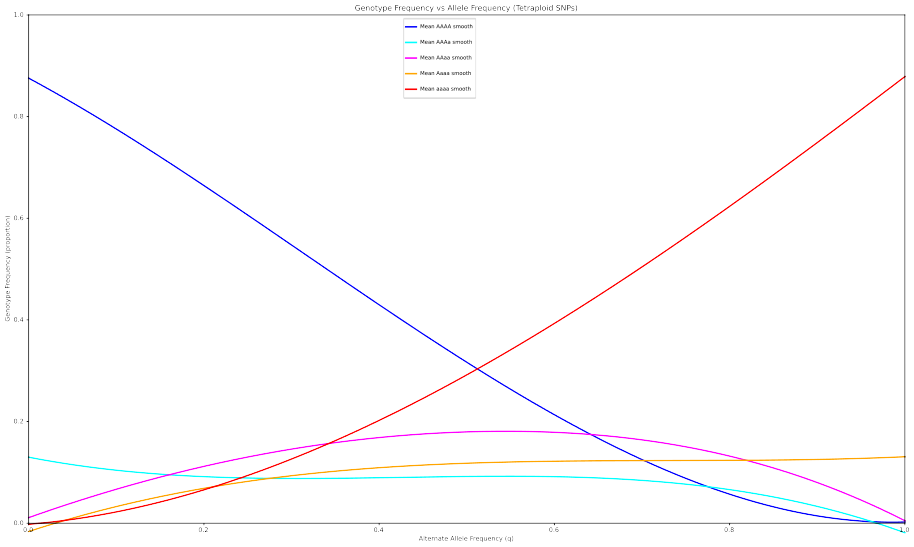
*

Unknown varieties (called diploids in GATK)

*
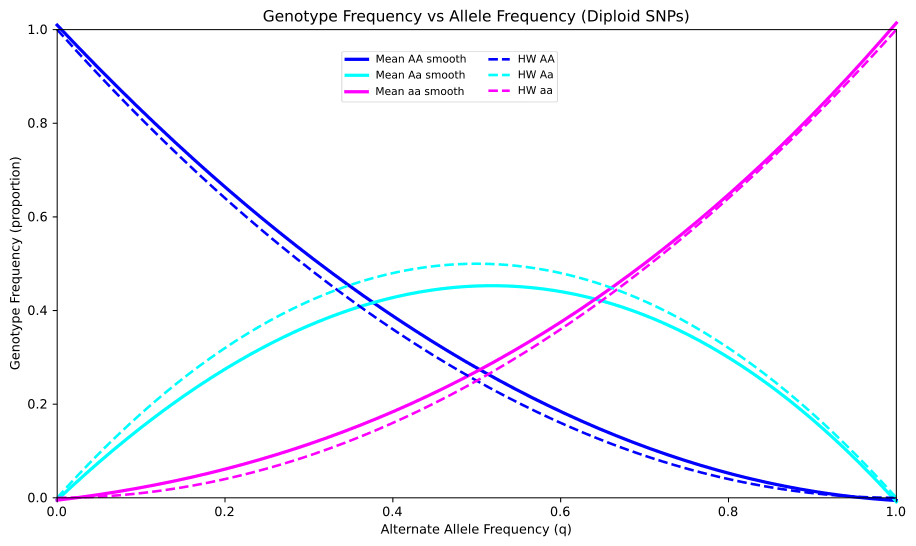
*
